# Artificial intelligence-aided method to detect uterine fibroids in ultrasound images: a retrospective study

**DOI:** 10.1038/s41598-022-26771-1

**Published:** 2023-03-06

**Authors:** Tongtong Huo, Lixin Li, Xiting Chen, Ziyi Wang, Xiaojun Zhang, Songxiang Liu, Jinfa Huang, Jiayao Zhang, Qian Yang, Wei Wu, Yi Xie, Honglin Wang, Zhewei Ye, Kaixian Deng

**Affiliations:** 1 Department of Orthopedics, Union Hospital, Tongji Medical College, Huazhong University of Science and Technology, Wuhan, China; 2 Department of Gynecology, Shunde Hospital, Southern Medical University (The First People’s Hospital of Shunde), Foshan, Guangdong China; 3 Research Institute of Imaging, National Key Laboratory of Multi-Spectral Information Processing Technology, Huazhong University of Science and Technology, Wuhan, China

**Keywords:** Machine learning, Ultrasonography

## Abstract

We explored a new artificial intelligence-assisted method to assist junior ultrasonographers in improving the diagnostic performance of uterine fibroids and further compared it with senior ultrasonographers to confirm the effectiveness and feasibility of the artificial intelligence method. In this retrospective study, we collected a total of 3870 ultrasound images from 667 patients with a mean age of 42.45 years ± 6.23 [SD] for those who received a pathologically confirmed diagnosis of uterine fibroids and 570 women with a mean age of 39.24 years ± 5.32 [SD] without uterine lesions from Shunde Hospital of Southern Medical University between 2015 and 2020. The DCNN model was trained and developed on the training dataset (2706 images) and internal validation dataset (676 images). To evaluate the performance of the model on the external validation dataset (488 images), we assessed the diagnostic performance of the DCNN with ultrasonographers possessing different levels of seniority. The DCNN model aided the junior ultrasonographers (Averaged) in diagnosing uterine fibroids with higher accuracy (94.72% vs. 86.63%, P < 0.001), sensitivity (92.82% vs. 83.21%, P = 0.001), specificity (97.05% vs. 90.80%, P = 0.009), positive predictive value (97.45% vs. 91.68%, P = 0.007), and negative predictive value (91.73% vs. 81.61%, P = 0.001) than they achieved alone. Their ability was comparable to that of senior ultrasonographers (Averaged) in terms of accuracy (94.72% vs. 95.24%, P = 0.66), sensitivity (92.82% vs. 93.66%, P = 0.73), specificity (97.05% vs. 97.16%, P = 0.79), positive predictive value (97.45% vs. 97.57%, P = 0.77), and negative predictive value (91.73% vs. 92.63%, P = 0.75). The DCNN-assisted strategy can considerably improve the uterine fibroid diagnosis performance of junior ultrasonographers to make them more comparable to senior ultrasonographers.

## Introduction

Uterine fibroids are by far the most common type of benign tumor in women^1^. Since CT and MRI examinations are costly and cannot be popularized, ultrasonography is currently the first imaging method used for the clinical diagnosis of uterine fibroids, as it possesses high sensitivity and specificity. Nevertheless, the following problems still exist in ultrasound uterine fibroid diagnoses. The first problem is the confusion between subplasmic and giant fibroids and between pelvic and adnexal masses^2^. Second, the current lack of standardized image acquisition views and the performance differences among types of ultrasound equipment impact the accuracy of fibroid detection. In addition, the accuracy of ultrasound uterine fibroid diagnosis depends on the knowledge and experience of ultrasonographers^3^.

Deep convolutional neural networks (DCNNs) based on deep learning (DL) techniques are new computer-aided diagnosis (CAD) tools that enable the automatic capture of targeted areas after training^4–9^. It is well-recognized by both researchers and clinicians that DL methods are already playing a substantial role in radiology because of their powerful image feature extraction capabilities^10^. DL algorithms for ultrasound image formation are rapidly garnering research support and attention, quickly becoming the latest frontier in ultrasound image formation^11^. A large number of previous studies on DCNN models based on ultrasound images mainly focused on the diagnosis of benign and malignant masses in superficial organs, such as the thyroid gland^12–15^ and breasts^16–19^. However, the diagnosis level of junior ultrasonographers with the assistance of this model has not only reached that of senior ultrasonographers but has also shortened the required diagnosis time. Deeper organs, such as the uterus, have been less studied. Therefore, it is necessary to develop a uterine fibroid detection DCNN model based on DL algorithms.

In this study, to improve the accuracy of ultrasound diagnosis for uterine fibroids, we developed a DCNN model that automates the detection of uterine fibroids in ultrasound images and discriminates between the presence and absence of fibroids and internally and externally validated the model. We also compared the DCNN model against eight ultrasonographers with different levels of seniority and explored whether the diagnostic performance of junior ultrasonographers can be improved with the assistance of this DCNN model.

## Methods

### Study patients and images

In this retrospective, noninterventional, case‒controlled study, we collected ultrasound images of patients who had been diagnosed with uterine fibroids according to surgical and pathological findings and those with normal uteri. Using these ultrasound images, we developed a DCNN model to automatically detect uterine fibroids in the images and to assist junior ultrasonographers in diagnosing uterine fibroids. To protect patients’ privacy, all identifying information, such as name, sex, age, and ID, on the ultrasound images was anonymized and omitted when the data were first acquired. This retrospective study was approved by the hospital ethics committee (Shunde Hospital of Southern Medical University). Additionally, all experiments were performed in accordance with relevant guidelines and regulations. All of the data in the study were obtained without any conflicts of interest.

A total of 3870 ultrasound images (2020 abnormal with uterine fibroids and 1850 normal) from 667 patients (mean age: 42.45 years ± 6.23 [SD]) with a pathological diagnosis of uterine fibroids and 570 women (mean age: 39.24 years ± 5.32 [SD]) without uterine lesions were included in the analysis. The DCNN was trained and developed on 3382 ultrasound images, which were randomly divided into a training dataset (80%, including 2706 images) and an internal validation dataset (20%, including 676 images). The model performance was tested on an external validation dataset containing 488 ultrasound images (268 uterine fibroids and 220 normal uteruses). The medical record system of the hospital provided us with the patients' clinical data (such as their ages, surgery statuses, pathological findings, surgical modalities, and postoperative ultrasound review findings). The information and imaging data of the patients with uterine fibroids are summarized in Tables 1 and 2, respectively. The proportion of uterine fibroids to normal uterine ultrasound images in each dataset is shown in Table 3.

**Table 1:** Clinical and pathological data summaries of the patients.

Variables	Value (n = 667)
Age (years), mean ± SD	42.45 ± 6.23
Diameter of uterine fibroids (cm), mean ± SD	6.14 ± 3.74
**Method of operation**	
Transabdominal	184 (27.6%)
Transvaginal	14 (2.1%)
Hysteroscopy	64 (9.6%)
Laparoscope	405 (60.7%)
**Surgical method**	
Removal of uterine fibroids	544 (81.6%)
Hysterectomy	123 (18.4%)
**Review**	
No	322 (48.3%)
Yes	345 (51.7%)
**Postoperative recurrence**	
Yes	102 (29.6%)
No	243 (70.4%)
**Pathology results**	
Non degeneration	576 (86.4%)
Red degeneration	18 (2.7%)
Glassy degeneration	61 (9.1%)
Cystic degeneration	1 (0.2%)
Sarcoma degeneration	0 (0.0%)
Fatty degeneration	0 (0.0%)
Calcification	11 (1.6%)

Except where indicated, the data are numbers of patients with percentages in parentheses. *SD* Standard deviation.

**Table 2:** Data summaries for the training, internal validation and internal validation test groups.

Variables	Training dataset (n = 1416)	Internal validation dataset (n = 336)	External validation dataset (n = 268)	*P* value
**Diameter of uterine fibroids (cm)**				0.53
< 4 cm	180 (12.7%)	37 (11.0%)	30 (11.2%)	
≥ 4 cm and < 8 cm	1038 (73.3%)	247 (73.5%)	191 (71.3%)	
≥ 8 cm	198 (14.0%)	52 (15.5%)	47 (17.5%)	
**Number of fibroids**				0.26
Single	680 (48.0%)	147 (43.8%)	134 (50%)	
Multiple ($\ge$ 2)	736 (52.0%)	189 (56.2%)	134 (50%)	
**Type of fibroids**				0.17
Sub serous fibroids	86 (6.1%)	13 (3.9%)	10 (3.7%)	
Intramural fibroids	1214 (85.7%)	303 (90.2%)	237 (88.4%)	
Submucosal fibroids	116 (8.2%)	20 (5.9%)	21 (7.8%)	
**Location of fibroids**				0.005
Cervix	11 (0.8%)	4 (1.2%)	5 (1.9%)	
Ante theca	377 (26.6%)	83 (24.7%)	58 (21.6%)	
Posterior	420 (29.7%)	134 (39.9%)	101 (37.7%)	
Fundus	136 (9.6%)	27 (8.0%)	25 (9.3%)	
The left side	187 (13.2%)	31 (9.2%)	24 (9.0%)	
The right side	169 (11.9%)	37 (11.0%)	34 (12.7%)	
Uterine cavity	116 (8.2%)	20 (6.0%)	21 (7.8%)	
**Type of ultrasound**				0.04
Transvaginal	1042 (73.6%)	267 (79.5%)	209 (78.0%)	
Abdominal	374 (26.4%)	69 (20.5%)	59 (22.0%)	

Except where indicated, the data are numbers of ultrasound images with percentages in parentheses.

**Table 3:** The proportion of uterine fibroids to normal uterine ultrasound images in each dataset.

	Training dataset	Internal validation dataset	External validation dataset	Total
Uterine fibroids	52% (1416/2706)	50% (336/676)	55% (268/488)	52% (2020/3870)
Normal uterine	48% (1290/2706)	50% (340/676)	45% (220/488)	58% (1850/3870)
Total	2706	676	488	3870

### Inclusion and exclusion criteria

The ultrasound images and clinical information data of patients with uterine fibroids and normal uteri were collected from Shunde Hospital of Southern Medical University between 2015 and 2020. We controlled the quality of the ultrasound images based on the associated pathological findings. The inclusion criteria for the abnormal patient group were as follows: (1) The preoperative ultrasound suggested the presence of a uterine mass; (2) no other combined uterine masses were present; (3) the ultrasound images were in black and white; and (4) the patients were diagnosed with uterine fibroids by two senior ultrasonographers (more than 10 years of clinical experience; 10 years of seniority or more). The exclusion criteria were as follows: (1) the patient lacked ultrasound data from our institution; and (2) images showing mass locations that did not match the clinical data. Finally, a total of 3870 ultrasound images (2020 uterine fibroids and 1850 normal uteruses) were acquired in this study.

### Image acquisition

All ultrasound images were acquired in.jpg format using a color Doppler ultrasound machine. The models included the APLI300 TUS-300, APLI400 TUS-400, APLI500 TUS-500, and LOGIQ S8. The operation routes were transabdominal or transvaginal, with the abdominal ultrasound probe set at 2–7 MHz and the vaginal ultrasound probe set at 5–7 MHz.

### Ground truth labeling

**Figure 1:**
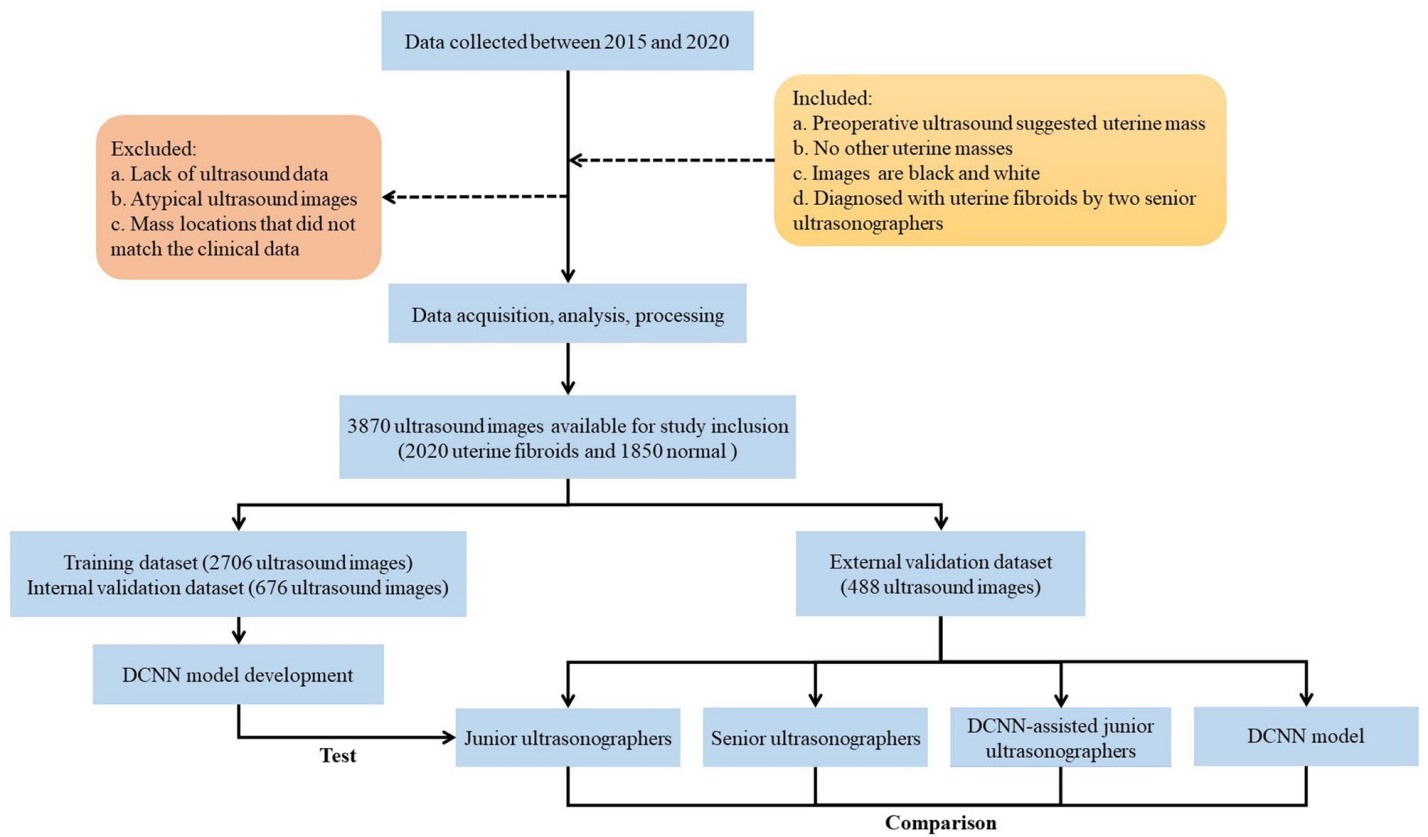
Flowchart of the procedures used in the development and evaluation of the DCNN model for automated uterine fibroid diagnosis. *DCNN* deep convolutional neural network.

**Figure 2:**
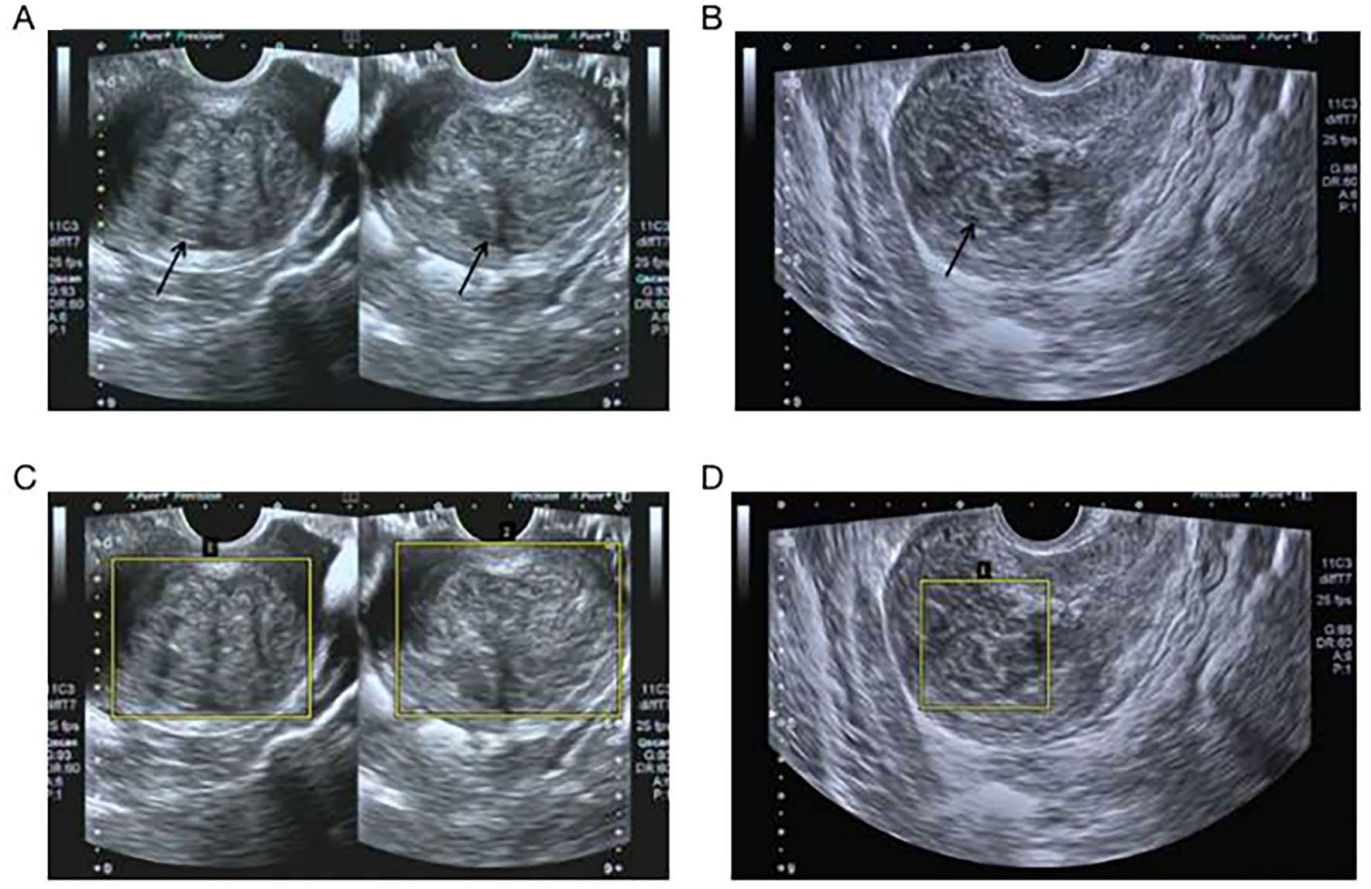
Examples of original ultrasound data and sample data after labeling. (**A**) Training sample of ultrasound image with two uterine fibroids. (**B**) Training sample of ultrasound image with one fibroid. (**C**) Labeled training sample of ultrasound image with two uterine fibroids. (**D**) Labeled training sample of an ultrasound image with only one fibroid. The yellow box shows the extent of fibroids marked by the ultrasonographers.

We randomly grouped the 3382 ultrasound images (1752 uterine fibroids and 1630 normal uteri) according to a training dataset (80%, 2706/3382) and an internal validation dataset (20%, 676/3382) for model training and development. The ground truths (GTs) of the training and validation datasets were labeled with the Visual Geometry Group Image Annotator software. Ultrasonographers with more than 10 years of experience labeled the ultrasound images of uterine fibroids through each patient’s clinical information and ultrasound image reports. If data samples with excessive labeling biases were generated, the final results were voted on again by the three ultrasonographers to determine the GT results. The system automatically generated json files, which included the image and information such, as the size and location of the GTs. The flow diagram is shown in Fig. 1. Examples of the original ultrasound data and sample data produced after labeling (including the GTs) are provided in Fig. 2.

### DCNN-based detection algorithm

In this study, we developed a two-stage DCNN model to detect uterine fibroids in ultrasound images. The network structure in this study consisted of two parts: (1) the YOLOv3^20^ detection network used to detect lesion regions in the ultrasound images and (2) another ResNet50^21^ network that was used to classify the images as normal or abnormal.

YOLOv3 is a state-of-the-art object detection network that uses features from the entire input image to predict a bounding box for each region of interest. ResNet50, a unique residual module, was used to replace the original network in YOLOv3 to learn more complex feature representations from the ultrasound images of uterine fibroids. The ResNet50 backbone was pretrained on the ImageNet^22^ classification task and fine-tuned on our training dataset. The original ultrasound images and the bounding boxes outlined by the radiologist, which covered the entire lesion area, were used as input data for training the YOLOv3 detection network.

**Figure 3:**
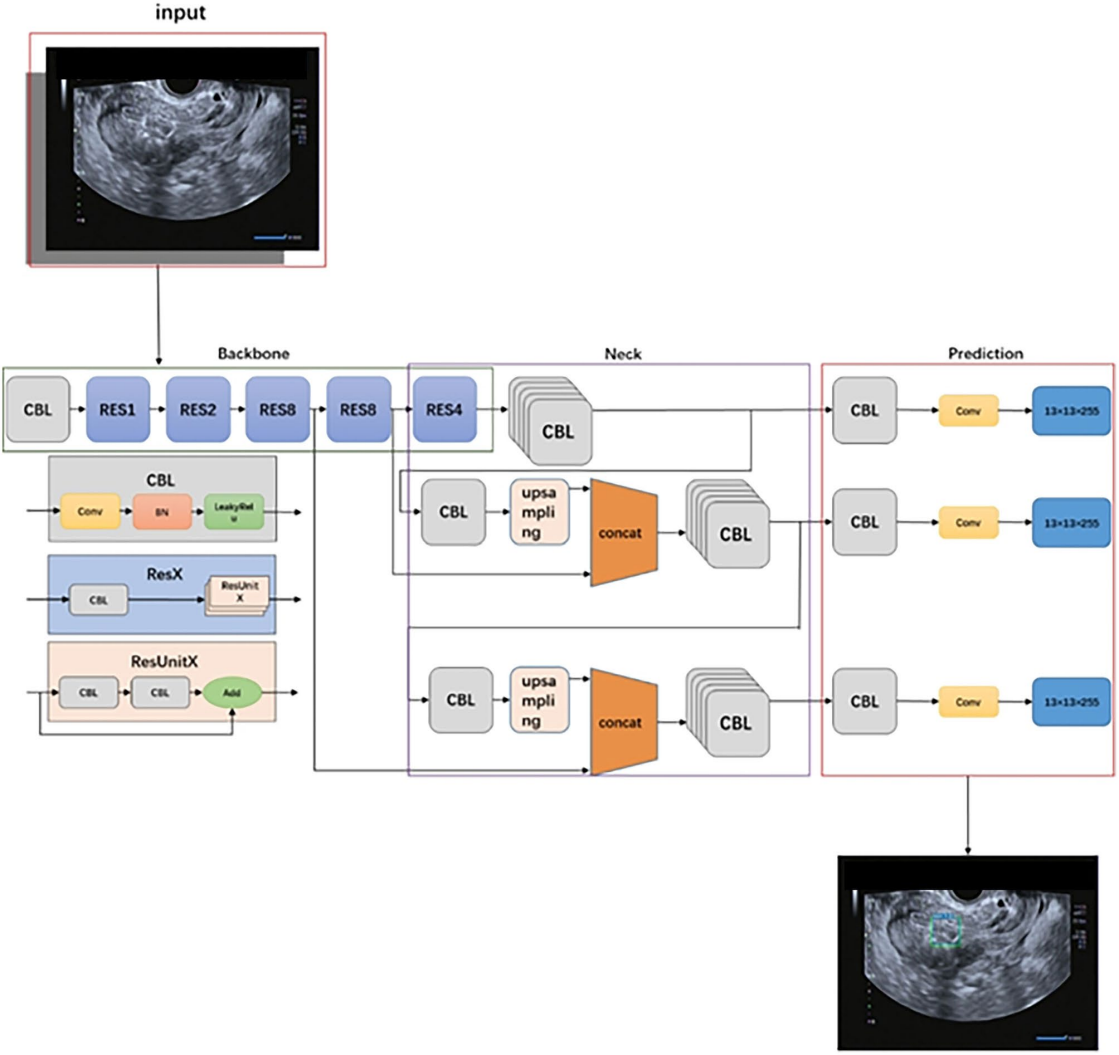
Outline of the DCNN for uterine fibroid detection. Schematic representation of the DCNN network architecture for uterine fibroid detection from ultrasound images. The CBL in front of each Res module acts as a downsampling. Con-cat = tensor concatenation, which expands the dimensionality of two tensors. Add: tensor summing, no dimensionality expansion. *Convs* Convolutional layers, *CBL* Minimum component in the structure of the YOLOv3 network united by the Conv + Bn + leaky-ReLU activation function. *Res-Unit* Residual structure, *Res-X* Large component in Yolov3 consisting of a CBL and X Res-Units.

**Figure 4:**
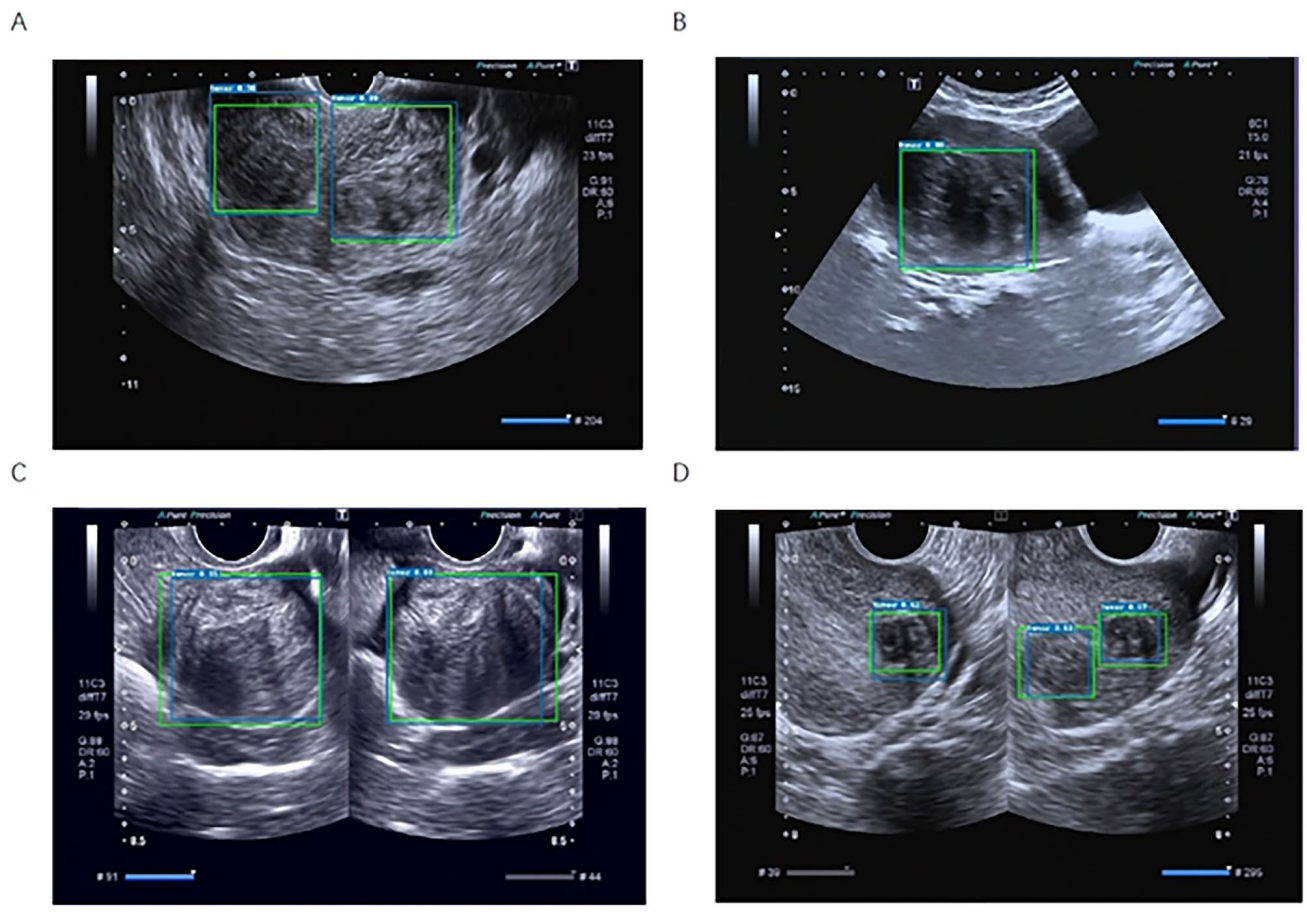
Uterine fibroid detection results. (**A**) Target detection results of multiple uterine fibroids in a single ultrasound image. (**B**) Target detection results of a single uterine fibroid in a single ultrasound image. (**C**) Target detection results of single uterine fibroids in patients with double ultrasound images. (**D**) Target detection results of multiple uterine fibroids in patients with double ultrasound images. Green boxes are the GT boxes annotated by our invited radiologists, and blue boxes are the boxes predicted by the trained model. The number marked in the upper left corner is the model detection confidence score.

All training and testing procedures were developed with Paddle (version 2.0.2), CUDA (version 10.1) and Python (version 3.7)^23^. Four graphics processing units (GPUs, NVIDIA GeForce GTX 1080Ti) were used, and the total training time was 10 h. The Adam^24^ optimizer was initialized, and each mini-batch contained 12 images. The weight decay was set as 0.0005, and the momentum was set as 0.9. A summary of the layers and output sizes of the ultrasound images produced by the DCNN during training is illustrated in Supplementary Table S1 online. The outline of the DCNN for uterine fibroid detection and some uterine fibroid detection results are shown in Figs. 3 and 4, respectively.

### Reference standard

In our local institution, the role of ultrasonographers is to examine the patient and make a diagnosis using a diagnostic ultrasound machine. In our study, four junior ultrasonographers (with less than 5 years of experience) and four senior ultrasonographers (with more than 10 years of experience) were selected to participate in determining the validation dataset, and none of them had previously been involved in the examination and interpretation of the validated ultrasound images. In the experiment, all identifying information, such as name, sex, age, and ID, on the ultrasound images were anonymized and omitted to ensure double-blindness. In addition, we scheduled the 8 ultrasonographers to perform the test at the same time in different locations to ensure their relative independence and temporal consistency. Each ultrasonographer independently interpreted the ultrasound image data contained in the external validation dataset and provided results, and four junior ultrasonographers interpreted the ultrasound image data again with the assistance of the DCNN model after interpreting the ultrasound image data individually.

### Statistical analysis

To compare the performance of DL methods with that of each ultrasonographer, we calculated their sensitivity, specificity, positive predictive value (PPV), negative predictive value (NPV) and accuracy metrics. Sensitivity was calculated as the percentage of correctly detected uterine fibroid images, and specificity was calculated as the percentage of correctly detected normal uterine images. The number of true positive (TP), false positive (FP), true negative (TN), and false negative (FN) findings yielded by the described methods were determined based on the pathological result. According to the TP rate (sensitivity) versus the FP rate (1-specificity), we calculated the areas under the receiver operating characteristic (ROC) curve and the 95% confidence interval (CI) for the junior ultrasonographers, the senior ultrasonographers, the DCNN model alone, and the DCNN-assisted junior ultrasonographers. Additionally, we used the pearson's chi-square test to test the difference in the interpretations of diagnosis of uterine fibroids between junior ultrasonographers and the DCNN model, senior ultrasonographers and the DCNN model, the DCNN-assisted junior ultrasonographers and senior ultrasonographers, junior ultrasonographers with and without the DCNN’s assistance, and *P* < 0.05 was considered a statistically significant difference. All statistical analyses were conducted using the software SPSS 22.0.

### Ethical approval

This study was performed in accordance with the Declaration of Helsinki and was approved by the hospital ethics committee (Shunde Hospital of Southern Medical University). All research was performed in accordance with relevant guidelines and regulations. We confirmed that informed consent was obtained from all participants and/or their legal guardians.

## Results

### The DCNN’s performance on the internal validation dataset

The diagnostic performance of the DCNN model was evaluated on an internal validation dataset of ultrasound images and compared with that of currently popular detection models. The intersections over union (IoUs, see Supplementary Fig. S1 online) and confidence thresholds were set to 0.5. For the internal validation dataset, the detection results of the DCNN model produced an F1 score of 0.94. We also found that the mean average precision (mAP) of the DCNN was 92.8%, and the time taken to read a single image was 162 ms (see Supplementary Table S2 online). A complete description of the image preprocessing method and the DCNN is provided in Supplementary Methods online. The same model developed with another architecture is provided in Supplementary Fig. S2 online.

### The DCNN performance compared with that of senior ultrasonographers and junior ultrasonographers

**Figure 5:**
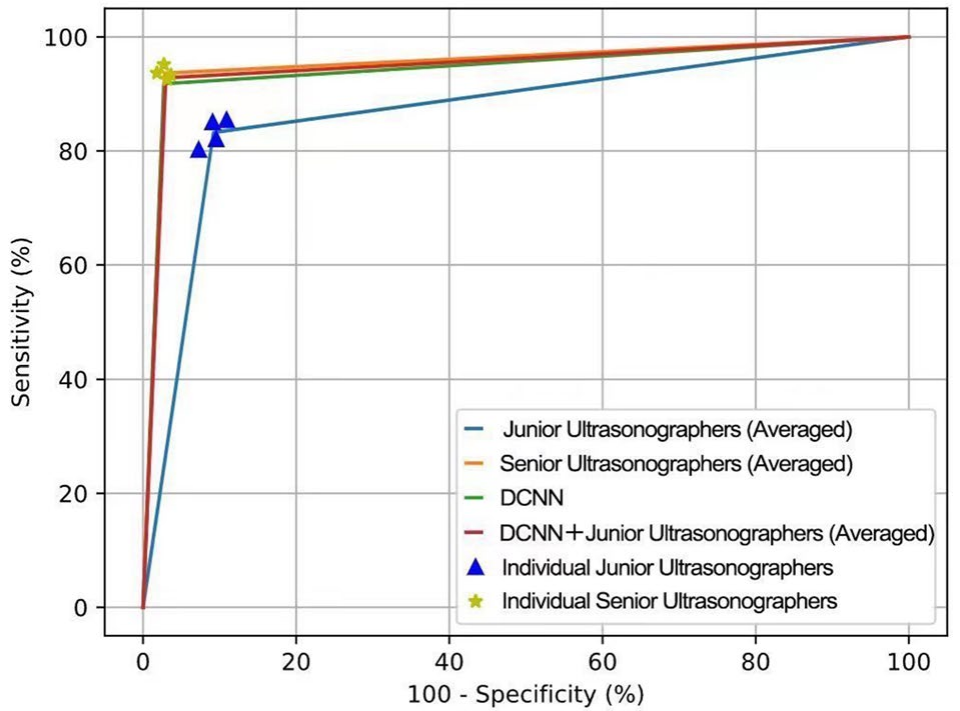
Receiver operating characteristic curves (ROC) show the diagnostic performance in uterine fibroids of the DCNN model, ultrasonographers with different seniority levels and junior ultrasonographers assisted by the DCNN model in the external validation dataset. The ROC curves for Junior ultrasonographers (Averaged) (blue), Senior ultrasonographers (Averaged) (orange), the DCNN model (green), and the DCNN + Junior ultrasonographers (Averaged) (red) provide corresponding AUCs of 0.87 (95% CI 0.84–0.91), 0.95 (95% CI 0.84–0.91), 0.95 (95% CI 0.92–0.97) and 0.95 (95% CI 0.93–0.97).

On the external validation dataset (268 uterine fibroids and 220 normal uteruses), we selected four junior ultrasonographers (with 5 years of experience or less) and four senior ultrasonographers (with more than 10 years of experience) to participate in this study. We compared the performance of the four junior ultrasonographers (Averaged), the four senior ultrasonographers (Averaged), the DCNN model and the four junior ultrasonographers (Averaged) with the DCNN-assisted in diagnosis of uterine fibroids, and the corresponding ROC graphs are shown in Table 4 and Fig. 5.

**Table 4:** Comparison between the DCNN model and ultrasonographers with different levels of seniority.

	Junior ultrasonographers (Averaged)	Senior ultrasonographers (Averaged)	DCNN model	DCNN + Junior ultrasonographers (Averaged)	P*^1^ value	P*^2^ value	P*^3^ value	P*^4^ value
PPV (%)	91.68 (223/243.25)	97.57 (251/257.25)	97.62 (246/252)	97.45 (247.75/255.23)	0.004	0.97	0.007	0.77
NPV (%)	81.61 (177.75/244.75)	92.63 (213.75/230.75)	90.68 (214/236)	91.79 (213.5/232.75)	0.004	0.44	0.001	0.75
Sensitivity (%)	83.21 (223/268)	93.66 (251/268)	91.79 (246/268)	92.82 (248.75/268)	0.003	0.41	0.001	0.73
Specificity (%)	90.80 (199.75/220)	97.16 (213.75/220)	97.27 (214/220)	97.05 (213.5/220)	0.005	> 0.99	0.009	0.79
Accuracy (%)	86.63 (422.75/488)	95.24 (464.75/488)	94.26 (460/488)	94.72 (462.25/488)	< 0.001	0.47	< 0.001	0.66
AUC	0.87 (95%CI 0.84–0.91)	0.95 (95%CI 0.92–0.97)	0.95 (95%CI 0.92–0.97)	0.95 (95%CI 0.93–0.97)				

*PPV* positive predictive value, *NPV* negative predictive value, *AUC* area under the receiver operating characteristic curve. P*^1^ value for Junior ultrasonographers (Averaged) compared to DCNN. P*^2^ value for Senior ultrasonographers (Averaged) compared to DCNN. P*^3^ value for DCNN + Junior ultrasonographers (Averaged) compared to Junior ultrasonographers (Averaged). P*^4^ value for DCNN + Junior ultrasonographers (Averaged) compared to Senior ultrasonographers (Averaged).

#### Comparison between the DCNN and ultrasonographers with different levels of seniority

On the external validation dataset, the DCNN model had higher accuracy (94.26% vs. 86.63%, P < 0.001), sensitivity (91.79% vs. 83.21%, P = 0.003), specificity (97.27% vs. 90.80%, P = 0.005), PPV (97.62% vs. 91.68%, P = 0.004), and NPV (90.68% vs. 81.61%, P = 0.004) than the junior ultrasonographers (Averaged). Moreover, the DCNN model had comparable accuracy (94.26% vs. 95.24%, P = 0.47), sensitivity (91.79% vs. 93.66%, P = 0.41), specificity (97.27% vs. 97.16%, P > 0.99), PPV (97.62% vs. 97.57%, P = 0.97), and NPV (90.68% vs. 92.63%, P = 0.44) to those of the senior ultrasonographers (Averaged).

#### Comparison of the junior ultrasonographers’ performance with and without the DCNN’s assistance

On the external validation dataset, the four junior ultrasonographers (with the assistance of the DCNN) showed substantial improvement in identifying the presence or absence of uterine fibroids. The DCNN model assisted junior ultrasonographers in diagnosing uterine fibroids with higher accuracy (94.72% vs. 86.63%, P < 0.001), sensitivity (92.82% vs. 83.21%, P = 0.001), specificity (97.05% vs. 90.80%, P = 0.009), PPV (97.45% vs. 91.68%, P = 0.007), and NPV (91.73% vs. 81.61%, P = 0.001) than they achieved alone. In terms of accuracy (94.72% vs. 95.24%, P = 0.66), sensitivity (92.82% vs. 93.66%, P = 0.73), specificity (97.05% vs. 97.16%, P = 0.79), PPV (97.45% vs. 97.57%, P = 0.77), and NPV (91.73% vs. 92.63%, P = 0.75), the DCNN-assisted junior ultrasonographers were comparable to the senior ultrasonographers (Averaged). Performance comparisons between the four junior ultrasonographers with and without the DCNN assistance are provided in Table 5.

**Table 5:** Performance comparisons between the four junior ultrasonographers with and without the DCNN’s assistance.

		Without DCNN	With DCNN	*P* value
Junior ultrasonographer A	Sensitivity (%)	85.07 (228/268)	94.03 (252/268)	0.001
	Specificity (%)	90.91 (200/220)	97.27 (214/220)	0.005
	Accuracy (%)	87.70 (428/488)	95.49 (468/488)	0.001
Junior ultrasonographer B	Sensitivity (%)	85.45 (229/268)	93.28 (250/268)	0.003
	Specificity (%)	89.09 (196/220)	96.36 (212/220)	0.003
	Accuracy (%)	87.09 (425/488)	94.67 (462/488)	< 0.001
Junior ultrasonographer C	Sensitivity (%)	80.22 (215/268)	91.42 (245/268)	< 0.001
	Specificity (%)	92.73 (204/220)	97.73 (215/220)	0.014
	Accuracy (%)	85.86 (419/488)	94.26 (460/488)	< 0.001
Junior ultrasonographer D	Sensitivity (%)	82.09 (220/268)	92.54 (248/268)	< 0.001
	Specificity (%)	90.45 (199/220)	96.82 (213/220)	0.006
	Accuracy (%)	85.86 (419/488)	94.47 (461/488)	< 0.001
Junior ultrasonographers (Averaged)	Sensitivity (%)	83.21 (223/268)	92.82 (248.75/268)	0.001
	Specificity (%)	90.80 (199.75/220)	97.05 (248.75/268)	0.009
	Accuracy (%)	86.63 (422.75/488)	94.72 (462.25/488)	< 0.001

## Discussion

In this study, we developed a DCNN model for the automated detection of uterine fibroids in ultrasound images and compared this DCNN with several current and more advanced DL detection models in terms of their uterine fibroid diagnosis performance on internal and external validation datasets. With the assistance of the DCNN model, the junior ultrasonographers performed better in diagnosing uterine fibroids than they did without the model, and the resulting diagnostic performance was comparable to that of senior ultrasonographers. In addition, the DCNN model was able to simultaneously and quickly detect multiple masses of different sizes in a single image.

To the best of our knowledge, this study is the first to evaluate a DL strategy as an aid for the diagnosis of uterine fibroids. In recent years, DL algorithms have been widely used in the field of medical image analysis^25–27^, and DCNN–based image detection methods have been commonly applied for classifying and identifying lesions^28–30^. However, no studies have been reported on the DCNNs in diagnosing uterine fibroids in ultrasound images and comparing with ultrasonographers thus far^31–33^. Even if artificial intelligence (AI) has superior performance, real world decisions should be supervised by ultrasonographers. Therefore, the most important role of an AI system is to assist junior ultrasonographers in improving the detectability of uterine fibroids.

The lack of both medical resources and senior ultrasonographers leads to poor uterine fibroid diagnostic performance and causes some patients to miss their optimal treatment opportunities^2^. However, the DCNN aids junior ultrasonographers in diagnosing uterine fibroids and is expected to improve this situation. The DCNN model developed in this study assisted junior ultrasonographers in diagnosing uterine fibroids in ultrasound images with higher sensitivity, specificity, and accuracy than they achieved alone. This suggests that the proposed DCNN AI model can be used to assist junior ultrasonographers in improving their diagnostic performance, thereby shortening the growth cycle of ultrasonographers.

This study has several limitations. First, this study is a single-center study, and the data may have selection bias^34^. In the future, multicenter studies should be conducted to improve the database required for training AI systems to improve the diagnostic capability of DCNN models. In addition, a combination of semisupervised and unsupervised network learning should be explored to reduce the data requirements of our model^35,36^. Second, we only trained and tested a single image of uterine fibroids instead of obtaining volume data from ultrasound images, such as CT or MRI. Selecting an image was itself influenced by the experience and skills of the performing ultrasonographers, but it does not mean that the images were purposely selected to be representative, only that there are factors that are objectively influenced. However, in real practice, ultrasonographers usually evaluate uterine fibroids using real-time ultrasound information, not an ultrasound image^37^. Thus, in the future, we need to study the real-time automated application of the DCNN or the DCNN with improved technology allowing the collection of ultrasound volume data, and we will gradually extend these studies to clinical work. Third, the current treatment plan for uterine fibroids is mainly based on clinical symptoms and does not rely exclusively on ultrasound diagnosis. Therefore, the DCNN model established in this study can only assist ultrasonographers in improving the diagnostic capability of uterine fibroids but not the clinical outcome of patients.

AI is unable to replace ultrasonographers by automating the diagnosis of diseases. However, as a tool for medical aid diagnosis^38^, it can reduce the burden of ultrasonographers in reading images. The proposed DCNN detection system can identify the presence of fibroids in ultrasound images, and it can also serve as a learning tool for junior ultrasonographers to learn to correctly differentiate uterine fibroids. In conclusion, the DCNN-assisted strategy considerably improved the uterine fibroid diagnostic performance of junior ultrasonographers. Further studies with larger sample sizes, enriched types of uterine masses, and generalized model structures are needed so that the proposed approach can be applied to other medical image analysis tasks in a quicker and more flexible manner.

## Supplementary Information

Supplementary Information.

## Data Availability

The datasets generated or analyzed during the study are available from the corresponding author on reasonable request. Access to the data will require that investigators provide a methodologically sound protocol, demonstrate that the ethics approval has been obtained by an accredited research ethics board, apply for ethics review at the Shunde Hospital, Southern Medical University (The First People's Hospital of Shunde) and sign a data sharing agreement.

## Abbreviations

AI: Artificial intelligence
DL: Deep learning
DCNN: Deep convolutional neural network
AUC: Area under the receiver operating characteristic curve
PPV: Positive predictive value
NPV: Negative predictive value
CT: Computed tomography
MRI: Magnetic resonance imaging
