# Supplementary material for: Artificial intelligence-aided method to detect uterine fibroids in ultrasound images: a retrospective study
Source: Sci Rep. 2023 Mar 6;13:3714. doi: 10.1038/s41598-022-26771-1 (PMC9988965; doi:10.1038/s41598-022-26771-1)
Supplement: Supplementary file 1 — Supplementary Information. [file 41598_2022_26771_MOESM1_ESM.pdf]

Artificial intelligence-aided method to detect uterine fibroids in ultrasound images: a retrospective study

Tongtong Huo, PhD[ac]*; Lixin Li, MS[b]*; Xiting Chen, MS[b]; Ziyi Wang, MR[c]; Xiaojun Zhang, MS[b]; Songxiang Liu, MD[a]; Jinfa Huang, MR[b]; Jiayao Zhang, MD[a]; Qian Yang, MS[b]; Wei Wu, MD[a]; Yi Xie, MD[a]; Honglin Wang, MR[a]; Zhewei Ye, MD[a]**; Kaixian Deng, MD[b]**

a Department of Orthopedics, Union Hospital, Tongji Medical College, Huazhong University of Science and Technology, Wuhan 430000, China;
b Department of Gynecology, Shunde Hospital, Southern Medical University (The First People's Hospital of Shunde), Foshan, Guangdong, 528308, China;
c Research Institute of Imaging, National Key Laboratory of Multi-Spectral Information Processing Technology, Huazhong University of Science and Technology, Wuhan, China.

[*]These authors contributed equally to this work.
[**]These authors contributed equally as cocorresponding authors.

Corresponding Author:
Prof Zhewei Ye, MD
Department of Orthopedics, Union Hospital, Tongji Medical College, Huazhong University of Science and Technology, Wuhan 430000, China
E-mail: yezhewei@hust.edu.cn
or
Prof Kaixian Deng, MD
Department of Gynecology, Shunde Hospital, Southern Medical University (The First People's Hospital of Shunde), Foshan, Guangdong, 528308, China

E-mail: nsyfek@163.com

Supplementary Information

1 Supplementary Methods

1.1 Model Developments

As an anchor-based and one-stage detection model, YOLOv3 has a faster detection speed than the other two-stage detectors, but the accuracy is not degraded too much. This is mainly due to the basic idea of its design.

YOLOv3 used an end-to-end architecture, which effectively utilizes the global. In addition, the author uses a deeper feature extraction network called darknet53 in YOLOv3, which contains batch normalization, a mixed residual network block and continuous 3 × 3 and 1 × 1 convolutions. Thus, the convergence effect is enhanced. It can directly predict the probability of an object category and the coordinates of its location, so the detection speed is relatively fast. However, it has a poor detection effect on small targets.

When the deep learning network layer is deeper, the expressive power is theoretically stronger. After the CNN reaches a certain depth, its classification performance will not be enhanced when its depth still deepens, but it will cause the network convergence to be slower and the accuracy rate to decrease. The network structure of ResNet can effectively solve the problem of gradient explosion and gradient disappearance caused by network depth. To more accurately detect relatively weak uterine fibroid targets in ultrasound images, we replaced the backbone darknet53 of YOLOv3 with ResNet50. The ResNet50 network structure is schematically shown in Table S1.

To the best of our knowledge, no algorithm has been applied to the detection of uterine fibroids. We compared our method with other well-known algorithms, trained each algorithm using our dataset, and compared the results with those of our method. To facilitate the comparison of performance differences between models, our DCNN model (YOLOv3 with ResNet50 backbone), referred to as YOLOv3+, was trained using the same dataset and tested on the same external validation set (268 ultrasound images containing uterine fibroids and 110 normal uterine images) with other well-known models [SSD, Faster RCNN, YOLO-101 (YOLOv3 with ResNet101 backbone), and YOLO-50 (YOLO with ResNet50 backbone)]. The detailed results are shown in Table S2.

1.2 Data Enhancement

Data enhancement was necessary to improve the generalizability and robustness of the developed network. We used the following methods to enhance the data in the experiment. We randomly zoomed and normalized the image size to between -0.5 and 0.5 and increased the hue of the image by -18 to 18 and the saturation, brightness and contrast by 0.5 to 1.5 with a probability of 0.5; 1) randomly turned the image left and right and randomly distorted the image; 2) the image was randomly expanded; the execution probability was 0.5, the maximum expansion ratio was 4, and the filling color values for expansion were R: 123.675, G: 116.28, and B: 103.53; 3) the image was cropped randomly; the ratio of the length to the width of the cropped area was 0.5 to 2, the effective intersection over union (IoUs) threshold was (0, 0.1, 0.3, 0.5, 0.7, 0.9), and the ratio of the cropped area to the original image was 0.3 to 1.

1.3 Machine Environment

All training and testing procedures were developed with Paddle (version 2.0.2), CUDA (version 10.1) and Python (version 3.7). Four graphics processing units (GPUs; NVIDIA GeForce GTX 1080Ti) were used, and the total training time was 10 hours. The Adam optimizer was initialized, and each mini-batch contained 12 images. The weight decay was set as 0.0005, and the momentum was set as 0.9.

We added different tricks to the image preprocessing, the structure of the backbone and the regression branch of the loss function to train the model. Compared with the original YOLOv3 network, our results are substantially improved. However, there are still many improvements in the algorithm for YOLOv3, such as optimization of target confidence and feature extraction.

1.4 Additional Architecture for the Development of a Deep Learning Model

The loss function of YOLOv3 is mainly composed of three parts: the coordinate loss of the prediction box, the classification loss of the target and the confidence loss of the category. We added different tricks to the regression branch of the loss function to train the model.

The model loss function consists of four parts:

1) Losses are determined based on the predicted center coordinates using the following loss function:

$$\lambda_{coord}\sum_{i=0}^{s^2}\sum_{j=0}^{B} l_{ij}^{obj}\,[(x_i-\hat{x_i})^2+(y_i-\hat{y_i})^2]$$

λ is a given constant that represents the weight of the loss. (x, y) is the actual position obtained from the training data, and $(\hat{x}, \hat{y}\ )$ is the position of the predicted bounding box. s^2 denotes the grid size. B stands for "box". l_{ij}^{obj} indicates that if the box at (i, j) has a target, l_{ij}^{obj} = 1; if there is no target in grid unit i, l_{ij}^{obj} = 0.

2) The width and height of the prediction bounding box are lost, and the following loss function is used:

$$\lambda_{coord}\sum_{i=0}^{s^2}\sum_{j=0}^{B} l_{ij}^{obj}\left[\left(\sqrt{\omega_i}-\sqrt{\widehat{\omega_i}}\right)^2+\left(\sqrt{H_i}-\sqrt{\widehat{H_i}}\right)^2\right]$$

ω and H indicate the width and height of the GT box, respectively.

3) The following loss function is used to determine the loss of the predicted category:

$$\sum_{i=0}^{s^2} l_{ij}^{obj}\sum_{j=0}^{B}\left[\left(p_i(c)-\widehat{p_i}(c)\right)^2\right]$$

4) A loss is formed based on the confidence of the prediction by using the following loss function:

$$\sum_{i=0}^{s^2}\sum_{j=0}^{B} l_{ij}^{obj}\left[\left(C_i-\widehat{C_I}\right)^2\right]+\lambda_{coord}\sum_{i=0}^{s^2}\sum_{j=0}^{B} l_{ij}^{noobj}\left[\left(C_i-\widehat{C_I}\right)^2\right]$$

Ci is the confidence score, and $\widehat{Ci}$ is the intersection of the predicted bounding box and the GT boxes when there is no object in grid cell, l_{ij}^{noobj}= 1; otherwise, l_{ij}^{noobj}= 0.

5) The total loss function is:

$$\boldsymbol{loss} = \boldsymbol{\lambda_{coord}} \sum_{i=0}^{s^2} \sum_{j=0}^{B} \boldsymbol{l_{ij}^{obj}} \left[(\boldsymbol{x_i} - \widehat{\boldsymbol{x_\iota}})^2 + (\boldsymbol{y_i} - \widehat{\boldsymbol{y_\iota}})^2 \right]$$

$$+ \boldsymbol{\lambda_{coord}} \sum_{i=0}^{s^2} \sum_{j=0}^{B} \boldsymbol{l_{ij}^{obj}} \left[\left(\sqrt{\boldsymbol{\omega_i}} - \sqrt{\widehat{\boldsymbol{\omega_\iota}}} \right)^2 + \left(\sqrt{\boldsymbol{h_i}} - \sqrt{\widehat{\boldsymbol{h_\iota}}} \right)^2 \right]$$

$$+ \sum_{i=0}^{s^2} \sum_{j=0}^{B} \boldsymbol{l_{ij}^{obj}} \left[\left(\boldsymbol{C_i} - \widehat{\boldsymbol{C_I}} \right)^2 \right] + \boldsymbol{\lambda_{coord}} \sum_{i=0}^{s^2} \sum_{j=0}^{B} \boldsymbol{l_{ij}^{noobj}} \left[\left(\boldsymbol{C_i} - \widehat{\boldsymbol{C_I}} \right)^2 \right]$$

$$+ \sum_{i=0}^{s^2} \boldsymbol{l_{ij}^{obj}} \sum_{j=0}^{B} \left[\left(\boldsymbol{p_i}(\boldsymbol{c}) - \widehat{\boldsymbol{p_\iota}}(\boldsymbol{c}) \right)^2 \right]$$

where λ is a given constant that represents the weight of the loss, and the highest penalty is given for the coordinate prediction (λ_{coord} = 5) with the lowest confidence prediction penalty when no target is detected (λ_{noobj}= 0.5).

The parameters of the model were set as follows:

An output layer and a fully connected layer of ResNet50 were modified according to our task. ResNet50 structure with the last layer replaced with a softmax layer and pretrained on ImageNet (a large-scale hierarchical image database) and then fine-tuned on our training set. We assigned different weights to each class when computing the loss to equalize the unbalanced datasets. Higher weights are assigned to classes with fewer images, and lower

weights are assigned to classes with more images. Batch normalization, learning rate decay and cross validation methods were used to reduce the risk of model overfitting. After trying different configurations, we obtained the best results using a batch size of 12, and stochastic gradient descent (SGD) was adopted to update the weights of the network. The IoU threshold was set to 0.5, and the confidence threshold was set to 0.5. The detection performance of each model was tested on 268 pieces of data containing uterine fibroids. The initial learning rate was 0.0005, and each minibatch contained four images. The AP was obtained by integrating the AUC. This task was single target detection, and AP=mAP at this time.

1.5 Model Fine-tuning

Fine-tuning is performed according to the following steps:

1. The neural network model with softmax layers of 1000 categories is pretrained on the source dataset (ImageNet dataset), i.e., the source model.

2. Create a new neural network model that replicates all the model designs and their parameters on the source model except the output layer, i.e., the target model.

3. Our goal is to perform 2 classifications, so the new softmax layer of the target model will consist of 2 categories instead of 1000 categories and randomly initialize the model parameters of the softmax layer.

4. Train the target model on the dataset of ultrasound images we collected. The output layer is trained from the head, while the parameters of the remaining layers are fine-tuned based on the parameters obtained from the source model.

1.6 Evaluation Indicators for the Comparison Experiments Between the Models

For the uterine fibroid detection model YOLOv3 in our study, the threshold is used as a limiting metric in several settings. Among them, the IoU threshold metric was used as a statistic for the number of TPs and FPs for calculating AP. The YOLOv3 model outputs multiple boxes when predicting a target; for example, there are various boxes corresponding to the same target. When IoU≥threshold, it is considered a true positive prediction box (TP: True-Positive). In contrast, if IoU<threshold, it is considered a false positive prediction (FP: False-Positive). Calculating the precision [precision=TP/(TP+FP)] and recall [recall=TP⁄(TP+FN)] of positive samples, we obtain the points on each P-R curve (with recall as the horizontal coordinate and precision as the vertical coordinate). By adjusting the threshold to vary from 0.5 to 0.95, we obtained the corresponding AP value under each threshold. The results of the model comparison showed that YOLOv3 has a substantial advantage over other detection models when IOU=0.5. In other words, YOLOv3 is faster and more accurate than the other models at this time. Then, we fixed the threshold U (0.5) of the IoU and changed the confidence threshold (0: 1: 0.1) to obtain the other points on the P-R curve. All points were connected to obtain the complete P-R curve, and the AP was calculated from the area under the P-R curve. To comprehensively and objectively evaluate the detection performance of each model, the experiment calculated and compared the following indicators:

IoU: This metric measures the degree of overlap between two detection frames (for target detection). The method of evaluating IoU is shown in Fig. S1.

$$\boldsymbol{IoU = {}^{Area\ of\ overlap\ (yellow\ box)}\Big/_{Area\ of\ union\ (red\ box)}}$$

By calculating the recall rate (recall), precision rate (precision), F1 score, average precision (AP) value, mean AP (mAP) index and output PR curve, the performance of each model was compared.

$$\boldsymbol{Precision = TP/(TP + FP)}$$

$$\boldsymbol{Recall = TP\ /\ (TP + FN)}$$

$$\boldsymbol{F1\ score = {}^{2 * P * R}/_{P + R}}$$

$$\boldsymbol{AP = \int_0^1 p(r)\, dr}$$

$$\boldsymbol{mAP = {}^{\sum_{i=1}^{k} AP_i}/_{K}\ (When\ K = 1, mAP\ =\ AP)}$$

1.7 Results

The experimental results show that the recall rate and accuracy of the YOLOv3+ model are 92.91% and 95.04%, respectively. Although the Faster RCNN model performs well in most detection tasks, it performs poorly in this task. It can be seen from the number of DRs that the Faster RCNN model is more suitable for multitarget detection tasks. In addition, the F1 score of the proposed model is 93.96%. which is substantially better than the second-highest detection model YOLOv3 (F1 score of 87.48%). YOLOv3 is commonly used for target detection in the medical field. The mAP (92.77%) of the proposed model is much higher than that of the other models, which proves that the model is more sensitive to fibroids and can detect uterine fibroids more accurately.

Although the YOLOv3+ model is slightly weak in computing speed, the test time on a single ultrasound uterine image is 162ms, which is slightly slower than the single-stage monitoring model SSD (73ms) and YOLOv3 (130ms), but it is also far beyond the average reading speed of professional doctors. Obviously, the unique residual structure of the model is an effective structure for detecting uterine fibroids. It can be observed that the mAP of YOLOv3+ was 92.77%, the mAP of YOLO-50 (green line) was 82.60%, that of YOLO-101 (purple line) was 81.14%, that of Faster R-CNN was 75.29%, that of SSD was 83.24% and that of YOLOv3 was 89.08%. In addition, the comparative ablation experiment, YOLOv3+, also confirmed the effectiveness of data enhancement to improve model performance. The experiment also showed that the F1 score of the proposed YOLOv3+ model reached 0.9396. The model mAP reached 92.77%, which was better than that of other methods, and the single-chip detection time cost was only 162ms (Fig. S2).

Compared with the other network, our results are considerably improved. However, there are still many improvements in the algorithm for YOLOv3, such as optimization of target confidence and feature extraction.

2 Supplementary Tables

Table S1. A schematic diagram of the ResNet50 network architecture

Layer name	**ResNet50**	ResNet101	VGG19	DarkNet53
Conv1_x	[7×7,64], stride2		[3×3,64]×2	$\begin{bmatrix}1\times1,32\\3\times3,64\end{bmatrix}\times1$
Conv2_x	3×3 Max pool, stride2			
	$\begin{bmatrix}1\times1,64\\3\times3,64\\1\times1,256\end{bmatrix}\times3$	$\begin{bmatrix}1\times1,64\\3\times3,64\\1\times1,256\end{bmatrix}\times3$	[3×3,256]×2	$\begin{bmatrix}1\times1,64\\3\times3,128\end{bmatrix}\times2$
Conv3_x	$\begin{bmatrix}1\times1,128\\3\times3,128\\1\times1,512\end{bmatrix}\times4$	$\begin{bmatrix}1\times1,128\\3\times3,128\\1\times1,512\end{bmatrix}\times4$	[3×3,256]×4	$\begin{bmatrix}1\times1,128\\3\times3,256\end{bmatrix}\times8$
Conv4_x	$\begin{bmatrix}1\times1,256\\3\times3,256\\1\times1,1024\end{bmatrix}\times6$	$\begin{bmatrix}1\times1,256\\3\times3,256\\1\times1,1024\end{bmatrix}\times23$	[3×3,512]×4	$\begin{bmatrix}1\times1,256\\3\times3,512\end{bmatrix}\times8$
Conv5_x	$\begin{bmatrix}1\times1,512\\3\times3,512\\1\times1,2048\end{bmatrix}\times3$	$\begin{bmatrix}1\times1,512\\3\times3,512\\1\times1,2048\end{bmatrix}\times3$	[3×3,512]×4	$\begin{bmatrix}1\times1,512\\3\times3,1024\end{bmatrix}\times4$
	Average pool,1000-D Fc, SoftMax		4096-dfc×2<br>1000-D Fc, SoftMax	

Note. The structures of ResNet50, ResNet101, VGG19 and DarkNet53 of different backbones. Conv_x = convolution layers. D = Dimensionality. Fc = Fully connected layer.

Table S2. Performance summary of deep learning models with respect to the detection of uterine fibroids

**Models**	**DR-box**	**Recall**	**Precision**	**F1 score**	**mAP**	**Time (ms)**
SSD	306	94	83	0.88	0.83	**73**
YOLOv3	251	85	90	0.87	0.89	130
Faster RCNN	403	88	66	0.75	0.75	302
**YOLOv3+**	252	93	**95**	**0.94**	**0.93**	162
YOLO-101	245	75	82	0.78	0.81	168
YOLO-50	308	**97**	82	0.89	0.83	157

Note. DR = The detection result.

3 Supplementary Figures

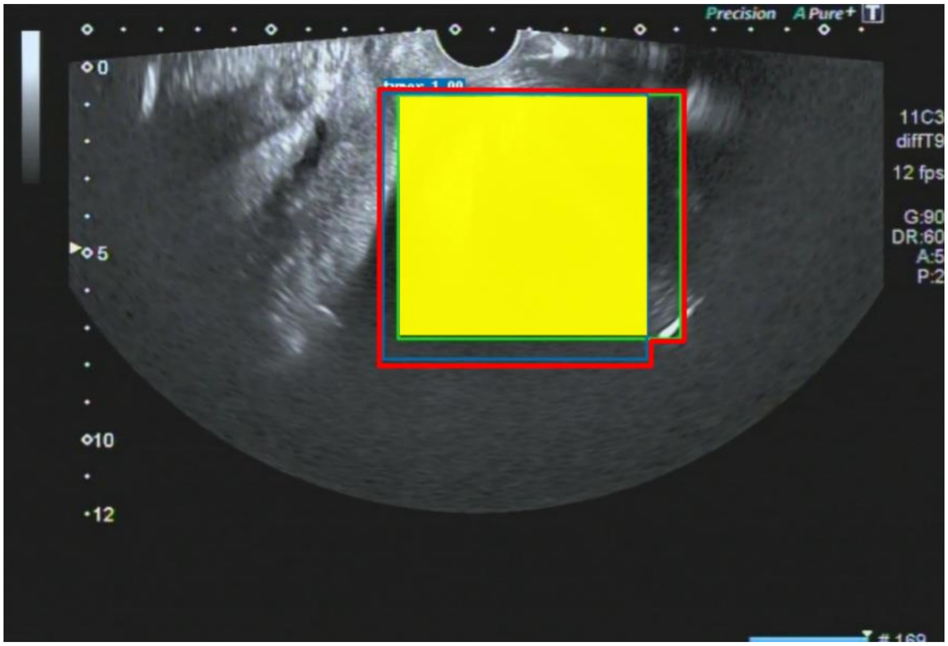

Figure S1. Schematic diagram of statistical indicators. The green box represents the GT. The blue box represents the detection result (DR). The yellow box represents the DR ∩ GT, which is the area of overlap. The red box represents the DR ∪ GT, which is the area of union. The indicators include true positives (TP: IoU ≥ 0.5 DR, one GT takes 1 DR); false positives (FP: IoU < 0.5, the remaining DR from the same GT); and false negatives (FN: samples with no uterine fibroids detected, FN = GT-TP) (Note: if the IoUs of multiple DR prediction boxes and the same GT box are greater than the threshold; then only the prediction box with the largest IoU is a TP, and the other is an FP).

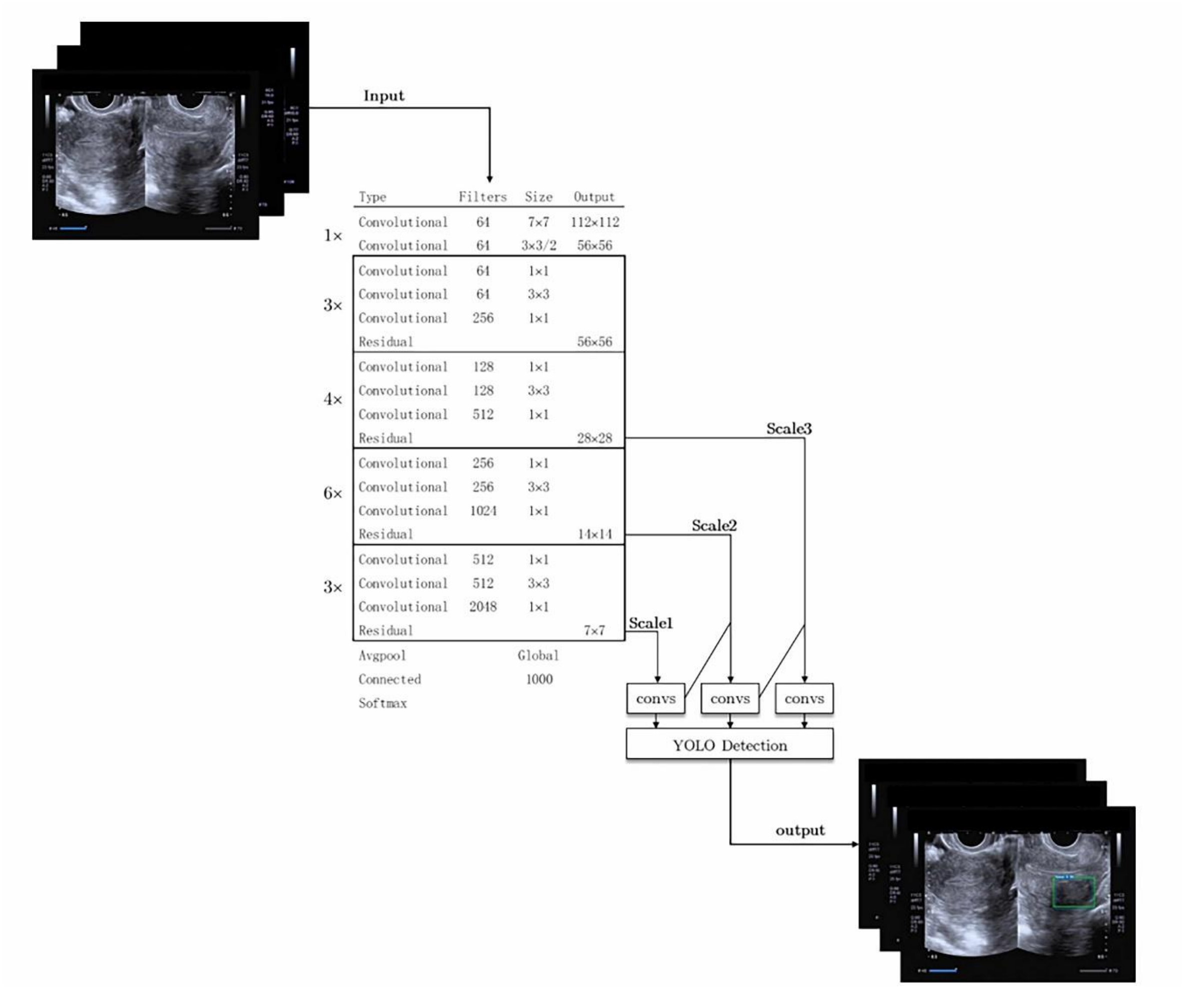

Figure S2. Outline of the DCNN model. The figure shows a schematic diagram of the DCNN network architecture for uterine fibroid detection from ultrasound images, in which ResNet50 is used as the feature extractor in the manuscript. Convs = convolution layers.
